# Abnormal Lysosomal Positioning and Small Extracellular Vesicle Secretion in Arterial Stiffening and Calcification of Mice Lacking Mucolipin 1 Gene

**DOI:** 10.3390/ijms21051713

**Published:** 2020-03-03

**Authors:** Owais M. Bhat, Xinxu Yuan, Sarah Camus, Fadi N. Salloum, Pin-Lan Li

**Affiliations:** 1 Department of Pharmacology and Toxicology, School of Medicine, Virginia Commonwealth University, Richmond, VA 23298, USA; Owais.Bhat@vcuhealth.org (O.M.B.); xinxu.yuan@vcuhealth.org (X.Y.); Sarah.Camus@vcuhealth.org (S.C.); 2 VCU Pauley Heart Center, Division of Cardiology, Department of Internal Medicine, Virginia Commonwealth University, Richmond, VA 23298-0204, USA; fadi.salloum@vcuhealth.org

**Keywords:** arterial medial calcification, *Mcoln1*^−/−^, multivesicular body, smooth muscle cells, small extracellular vesicle

## Abstract

Recent studies have shown that arterial medial calcification is mediated by abnormal release of exosomes/small extracellular vesicles from vascular smooth muscle cells (VSMCs) and that small extracellular vesicle (sEV) secretion from cells is associated with lysosome activity. The present study was designed to investigate whether lysosomal expression of mucolipin-1, a product of the mouse *Mcoln1* gene, contributes to lysosomal positioning and sEV secretion, thereby leading to arterial medial calcification (AMC) and stiffening. In *Mcoln1*^−/−^ mice, we found that a high dose of vitamin D (Vit D; 500,000 IU/kg/day) resulted in increased AMC compared to their wild-type littermates, which was accompanied by significant downregulation of SM22-*α* and upregulation of RUNX2 and osteopontin in the arterial media, indicating a phenotypic switch to osteogenic. It was also shown that significantly decreased co-localization of lysosome marker (Lamp-1) with lysosome coupling marker (Rab 7 and ALG-2) in the aortic wall of *Mcoln1*^−/−^ mice as compared to their wild-type littermates. Besides, *Mcoln1*^−/−^ mice showed significant increase in the expression of exosome/ sEV markers, CD63, and annexin-II (AnX2) in the arterial medial wall, accompanied by significantly reduced co-localization of lysosome marker (Lamp-1) with multivesicular body (MVB) marker (VPS16), suggesting a reduction of the lysosome-MVB interactions. In the plasma of *Mcoln1*^−/−^ mice, the number of sEVs significantly increased as compared to the wild-type littermates. Functionally, pulse wave velocity (PWV), an arterial stiffening indicator, was found significantly increased in *Mcoln1*^−/−^ mice, and Vit D treatment further enhanced such stiffening. All these data indicate that the *Mcoln1* gene deletion in mice leads to abnormal lysosome positioning and increased sEV secretion, which may contribute to the arterial stiffness during the development of AMC.

## 1. Introduction

Vascular calcification (VC) was considered to be a passive and degenerative process, whereas now it is considered to be an actively regulated process associated with the pathological deposition of calcium minerals in the vascular system [1,2]. The intimal calcification is associated with an increased deposit of lipids, and inflammatory cell infiltrate, while medial calcification involves a phenotypic transformation of vascular smooth muscle cells (VSMCs) in the arterial media [3,4]. Medial calcification is commonly found in patients with advanced age, diabetes, calcific aortic valve disease, chronic kidney disease (CKD), and chronic inflammatory diseases [5]. One of the possible mechanisms of VC is phenotypic change of VSMCs from a contractile to a synthetic and osteochondrogenic phenotype followed by augmented expression of osteochondrogenic markers, for example, osteocalcin, osteopontin, RUNX2, and alkaline phosphatase and reduced expression of VSMC contractile markers such as smooth muscle *α*-actin and smooth muscle 22*α* (SM22-*α*). Another proposed mechanism is the release of matrix vesicles from viable VSMCs to initiate the VC [6]. 

Exosomes (40–140 nm in size) are a group of extracellular vesicles (EVs) with intracellular contents, such as proteins and RNAs [7] that are associated with VC [8,9]. Exosomes are secreted by VSMCs in vivo after pro-calcifying stimulation and become calcified to initiate VC [8]. Recent investigations revealed that an increase in exosomes secreted by VSMCs promotes VC via mineral deposition. Additionally, exosomes derived from calcified VSMCs were demonstrated to enhance calcification in the recipient VSMCs through initiating mitogen-activated protein kinase [10]. It is reported that the exosome secretion pathway is activated during VC, and modulations on such a pathway may be applied as novel approaches for VC prevention [8]. During VC progression, the phenotypic transition process involves cytoskeleton remodeling, such as intracellular modifications, which enhanced exosome secretion [11,12]. 

Lysosome coupling or trafficking is a crucial process to perform the lysosome fusion and fission events [13,14]. Various proteins are involved in lysosome coupling such as dynein-dynactin, Rab proteins, ALG-2, Arl8, etc., which in association with SNARE proteins are essential for the fusion/fission process. Rab7, a small GTPase, and lysosomal Ca^2+^-sensor ALG-2 (also known as PDCD6) are known to play essential roles in the lysosome coupling process [15]. Belonging to the large family of transient receptor potential ion channels, lysosome Transient receptor potential mucolipin 1 (encoded by the *Mcoln1* gene, also known as TRPML1) [16,17] permeates Ca^2+^, Na^+^, and other cations [18,19,20]. Mutations in the *TRPML1* gene lead to mucolipidosis type IV (ML4) disease in humans, which is characterized by defects in membrane trafficking in the late endocytic pathway due to enlarged lysosomes [16,21] and impaired lysosome biogenesis [22,23]. In TRPML1^−/−^ osteoclasts, markedly enlarged lysosomes were observed as occurring in lysosomal storage diseases [24], which lead to defective lysosomal trafficking. 

It has been reported that TRPML1 plays a critical role in lysosomal trafficking and various other lysosomal functions [25,26]. Mucolipin 1, also known as TRPML1, in association with Rab7, ALG-2, and other proteins, regulates centripetal transport of lysosomes crucial for lysosome exocytosis in response to changes in Ca^2+^ levels [15]. TRPML1 induces perinuclear redistribution of lysosomes, then links with ALG-2, which consequently causes TRPML1-dependent retrograde transport of lysosomes [27]. Based on earlier studies, multivesicular bodies (MVBs) and lysosome fusion are critical for the lysosomal degradation of MVBs [28,29]. Recently, a study and ours, also demonstrated that TRPML1 regulates lysosome fusion with MVBs and the plasma membrane which contributes to exosome release [30]. Our findings in podocytes demonstrated that inhibition or activation of the TRPML1 channel modulates exosome release from these cells [31]. Recently, we observed that inhibition of the TRPML1 channel by increased production of lysosomal sphingolipids due to acid ceramidase deficiency in arterial smooth muscle cells (SMCs) prevented lysosome-MVB interactions which resulted in enhanced small extracellular vesicle (sEV) secretion triggering arterial medial calcification (AMC) (unpublished data). Although, there is no direct evidence about whether *Mcoln1* is associated with arterial stiffness in vivo. Several studies revealed that transient receptor potential (TRP) channels contribute to the VSMC phenotypic transition and vasoconstriction, also release of calcified vesicles from these VSMCs [8,32], which are features that are involved in arterial stiffness during the progression of AMC [33]. 

Based on this knowledge, the present study investigated whether *Mcoln1* gene deletion enhances phenotypic switch in arterial medial SMCs in a mouse model of AMC. We further explored whether *Mcoln1* gene deletion contributes to impaired lysosome positioning-induced sEV secretion in arterial medial SMCs, which may implicate a mechanistic pathway leading to AMC and arterial stiffening. Our data suggest that *Mcoln1* deficiency impairs lysosomal positioning, which may prevent lysosome-MVB interaction and increase sEV secretion. Besides, we also determined that the *Mcoln1* gene contributes to phenotype change and arterial stiffness in vivo.

## 2. Results

### 2.1. Characterization of Mucolipin 1 (Mcoln1) Gene KO Mice

**Figure 1:**
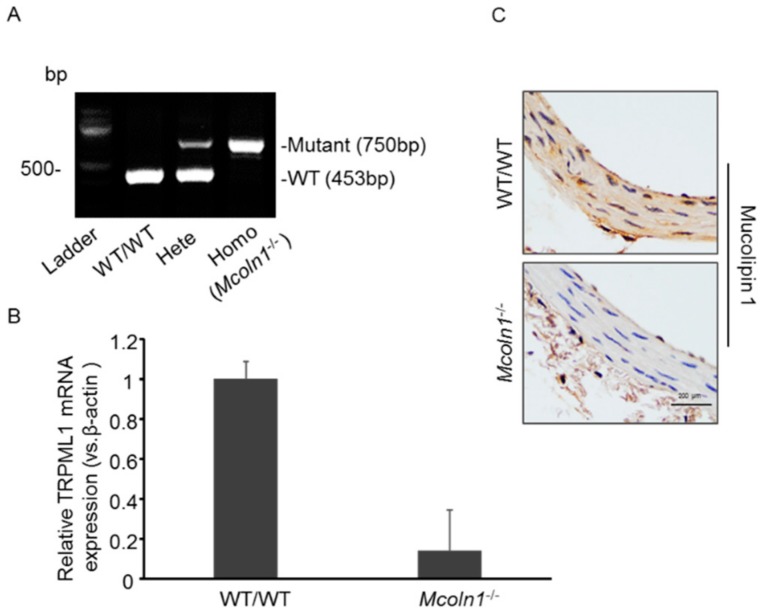
Characterization of *Mcoln1*^−/−^ mice. (**A**) Mucolipin 1 KO (Homozygous, *Mcoln1*^−/−^) had one positive PCR product for mutant 750 bp. Heterozygous mice had two positive PCR products including 750 bp for mutant and 453 bp for WT/WT. WT/WT mice only had wild-type *Mcoln1* gene (453 bp). (**B**) *Mcoln1* gene deletion was validated by RT-PCR as depicted by mRNA expression of *TRPML1* gene, also known as *Mcoln1*. Data are shown as mean ± SEM of values. *n* = 3. (**C**) Representative immunohistochemical images of aortic sections reveal abolished immunostaining of mucolipin 1 (brown stain) in *Mcoln1*^−/−^ mice as compared to wild-type. *n* = 3. bp: Base Pair; Homo: homozygous; Hete: heterozygous.

To characterize the mice, polymerase chain reaction (PCR) was used to verify the mice homozygous for the *Mcoln1* gene deletion. As shown in Figure 1A, *Mcoln1*^−/−^ has only mutant 750 bp PCR product while the heterozygous has both the mutant 750 bp and WT 453 bp PCR products. Wild-type mice had only the 453 bp PCR product. RNA was isolated from aortic tissues to analyze the mRNA expression of TRPML1, also known as *Mcoln1* gene, in both wild-type and *Mcoln1*^−/−^ mice. RT-PCR shows *Mcoln1* gene deletion in *Mcoln1*^−/−^ mice as compared to wild-type mice, as shown in Figure 1B. Immunostaining was used to confirm the expression of mucolipin 1 in aortic sections to validate *Mcoln1* gene deletion; we found positive immunostaining of mucolipin 1 in WT/WT mice, which was absent in *Mcoln1*^−/−^ mice as shown in Figure 1C. 

### 2.2. Mcoln1 Gene Deletion Enhanced Arterial Medial Calcification in Vit D-Treated Mice

**Figure 2:**
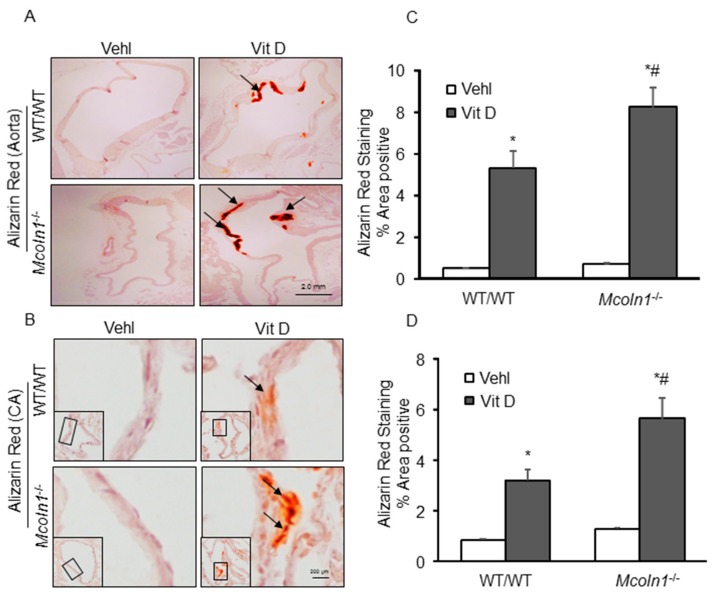
Arterial calcification in Vit D-treated *Mcoln1*^−/−^ mice. Representative images of (**A**) Aortic and (**B**) Coronary artery sections stained by Alizarin Red S (red color) staining show significantly increased calcification in the aortic (*n* = 5–6) and coronary arterial (*n* = 5) medial wall of Vit D-treated *Mcoln1*^−/−^ mice as compared to their littermates. (**C**,**D**) Summarized data showing calcification in the aortic and coronary arterial media. Data are shown as mean ± SEM of values. Vehl: Vehicle; Vit D: vitamin D; CA: coronary artery. * *p* < 0.05 vs. WT/WT Vehl; # *p* ˂ 0.05 vs. WT/WT Vit D group.

To investigate the role of *Mcoln1* gene in AMC in vivo, subcutaneous (s.c.) injections of vitamin D (Vit D) were given to mice for four consecutive days. Calcium deposition was visualized in aortic cross sections by Alizarin Red staining. As shown in Figure 2A, representative images show markedly increased calcium deposition as reddish areas in the aortic media wall of *Mcoln1*^−/−^ mice as compared to their wild-type littermates. Moreover, we found significant changes in plasma calcium levels among treatments (WT and *Mcoln1*^−/−^ vehicle ~2.9 Mm, and WT and *Mcoln1^−/−^* Vit D ~3.69 mM) but there was a non-significant increase among genotypes (WT and *Mcoln1*^−/−^ mice). Furthermore, in coronary arteries, the photomicrographs show prominently increased calcium deposition due to *Mcoln1* gene deletion in *Mcoln1*^−/−^ mice as compared to wild-type mice, as shown in Figure 2B. The summarized data in bar graph form are shown in Figure 2C,D. These results indicated that *Mcoln1* gene contributes to the AMC.

### 2.3. Arterial Medial SMC Phenotypic Transition in Vit D-Treated Mcoln1^−/−^ Mice

**Figure 3:**
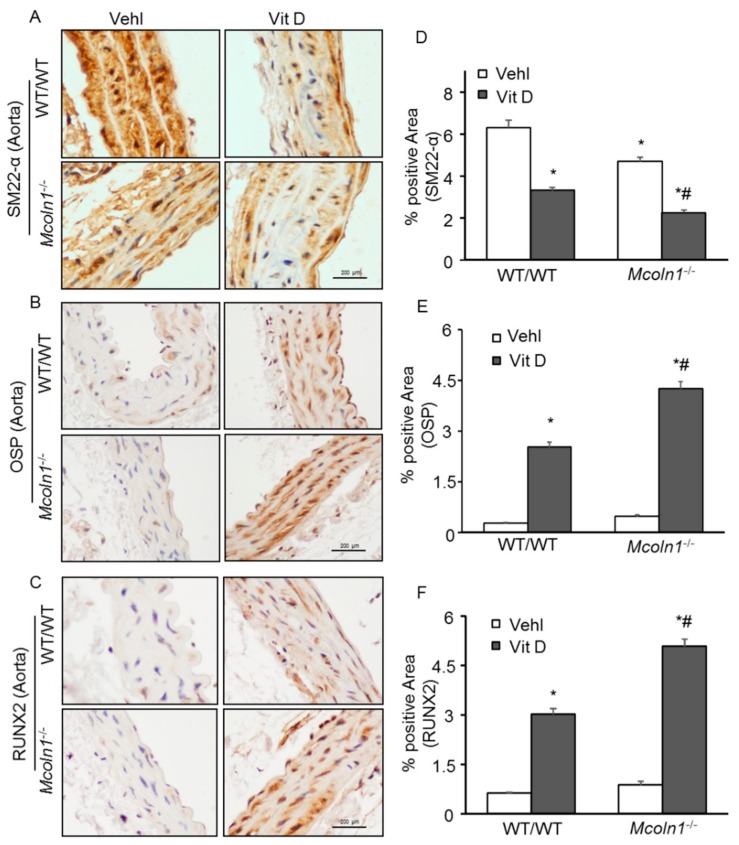
Phenotypic switch of smooth muscle cells (SMCs) in the aortic media in Vit D-treated *Mcoln1*^−/−^ mice. Representative aortic photomicrographs depict arterial medial calcification (AMC) associated with (**A**,**D**) decreased expression of SM22-α (brown stain) (*n* = 5–6); (**B**,**E**) increased expression of OSP (*n* = 6); and (**C**,**F**) RUNX2 (*n* = 6) (brown stain) in the aortic media of *Mcoln1*^−/−^ mice receiving Vit D injections as compared to wild-type littermates. Data are shown as mean ± SEM of values. Vehl: Vehicle; Vit D: vitamin D; OSP: Osteopontin; RUNX2: Runt-related transcription factor 2. * *p* ˂ 0.05 vs. WT/WT Vehl; # *p* ˂ 0.05 vs. WT/WT Vit D group.

Smooth muscle cells are involved in vascular calcification and their phenotypic change from contractile to osteogenic is depicted by the development of mineralization, calcification prone matrix downregulation of SMC markers (SM22*α* and SM *α*-actin), and upregulation of osteochondrogenic markers such as RUNX2, osteopontin (OSP), osteocalcin, alkaline phosphatase (ALP) [34], etc. To determine whether *Mcoln1* gene deletion is associated with phenotype change of SMC to osteogenic in AMC, immunostaining for SM22*α*, OSP, and RUNX2 was performed in the aortic sections. Under vehicle conditions, we observed a non-significant decrease in SMC contractile marker SM22-*α* in the aortic medial wall of *Mcoln1*^−/−^ mice as compared to their wild-type littermates, which was significantly decreased within the calcified areas after receiving Vit D treatment (Figure 3A,D). Moreover, the osteogenic markers such as OSP (Figure 3B) and RUNX2 (Figure 3C) were markedly increased in the arterial medial wall of Vit D-treated *Mcoln1*^−/−^ mice compared with their wild-type littermates. The summarized data are shown as bar diagrams (Figure 3E,F). 

**Figure 4:**
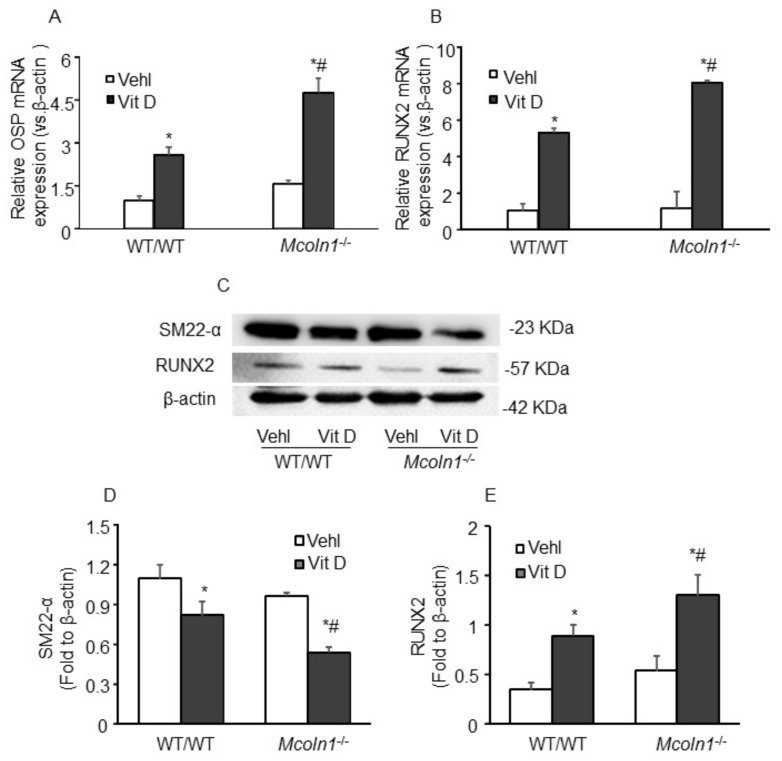
mRNA and protein expression of phenotypic switch markers in Vit D-treated *Mcoln1*^−/−^ mice. The bar graph shows significantly increased aortic mRNA expression of osteogenic markers (**A**) OSP and (**B**) RUNX2 in *Mcoln1*^−/−^ mice receiving Vit D injections as compared to wild-type littermates. *n* = 3–4. (**C**) Representative Western blot analysis showing the effects of *Mcoln1* gene deletion on SM22-*α* and RUNX2 protein expression. Summarized data show the changes in the protein expression of (**D**) SM22-*α* and (**E**) RUNX2. (*n* = 3–4). Data are shown as mean ± SEM of values. Vehl: Vehicle; Vit D: vitamin D; OSP: Osteopontin; RUNX2: Runt-related transcription factor 2. * *p* ˂ 0.05 vs. WT/WT Vehl; # *p* ˂ 0.05 vs. WT/WT Vit D group.

**Figure 5:**
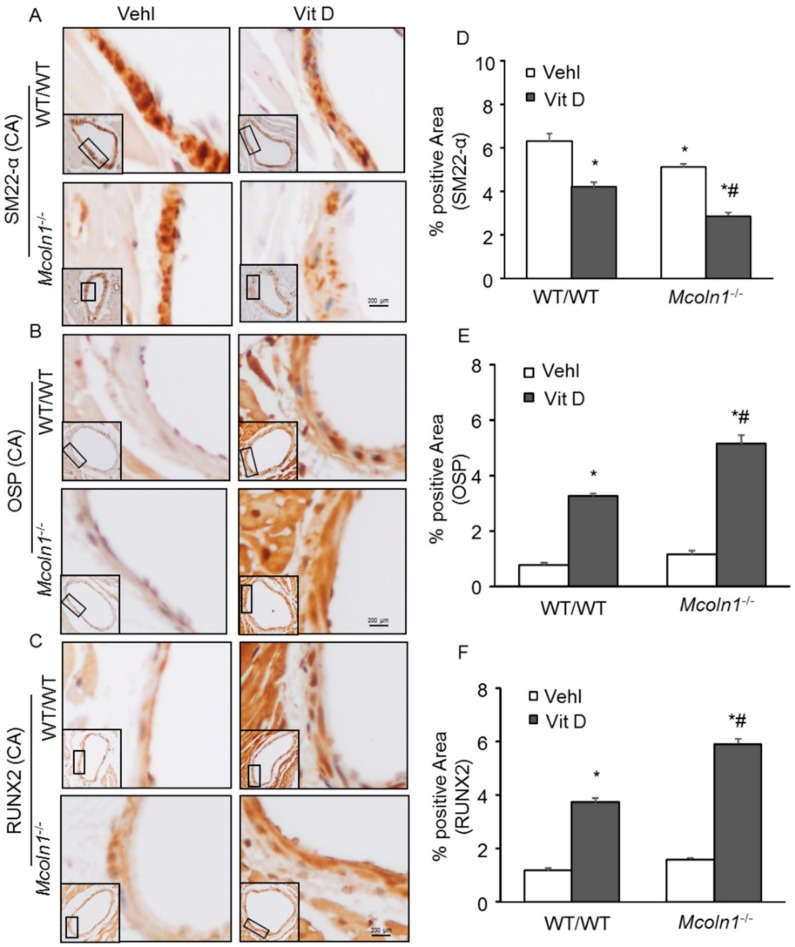
Phenotypic switch of SMCs in the coronary arterial wall in Vit D-treated *Mcoln1*^−/−^ mice. Representative immunohistochemical images of coronary artery sections show decreased expression of (**A**) SM22-*α* (brown stain) (*n* = 5) and increased expression of (**B**) OSP (*n* = 5); and (**C**) RUNX2 (*n* = 5) (brown stain) in the coronary arterial wall of Vit D-treated *Mcoln1*^−/−^ mice as compared to their littermates (**D**–**F**). The data of SMC phenotypic switch are summarized in the bar graph. Data are shown as mean ± SEM of values. Vehl: Vehicle; Vit D: vitamin D; SM22-*α*: smooth muscle cell marker; OSP: Osteopontin; RUNX2: Runt-related transcription factor 2. * *p* ˂ 0.05 vs. WT/WT Vehl; # *p* ˂ 0.05 vs. WT/WT Vit D group.

In addition, RNA was isolated from aortic tissues and, using RT-PCR, we demonstrated that *Mcoln1* gene deletion induced augmented mRNA expression of OSP and RUNX2 in Vit D-treated mice, as shown in Figure 4A,B. Also, Western blot analysis (Figure 4C) from aortic tissue protein lysates showed significant decrease in protein expression of SM22-*α,* while an increased expression of RUNX2 was observed as shown in the bar graph (Figure 4D,E). The changes were more prominent with *Mcoln1* gene deletion in Vit D-treated *Mcoln1^−/−^* mice as compared to WT/WT mice. These results revealed that *Mcoln1* gene might be associated with phenotype change of arterial medial SMCs during the development and progression of AMC. Moreover, we observed decreased immunostaining of SM22*α* (Figure 5A) in the coronary arterial wall associated with increased expression of OSP (Figure 5B) and RUNX2 (Figure 5C) in Vit D treated *Mcoln1*^−/−^ mice as compared to their wild-type littermates, as shown in Figure 5D–F. 

### 2.4. ALG-2 and Rab7 Interact with the Lysosome to Mediate Mcoln1-Dependent Lysosome Coupling in Aortic Medial SMCs in Vit D-Treated Mcoln1^−/−^ Mice

**Figure 6:**
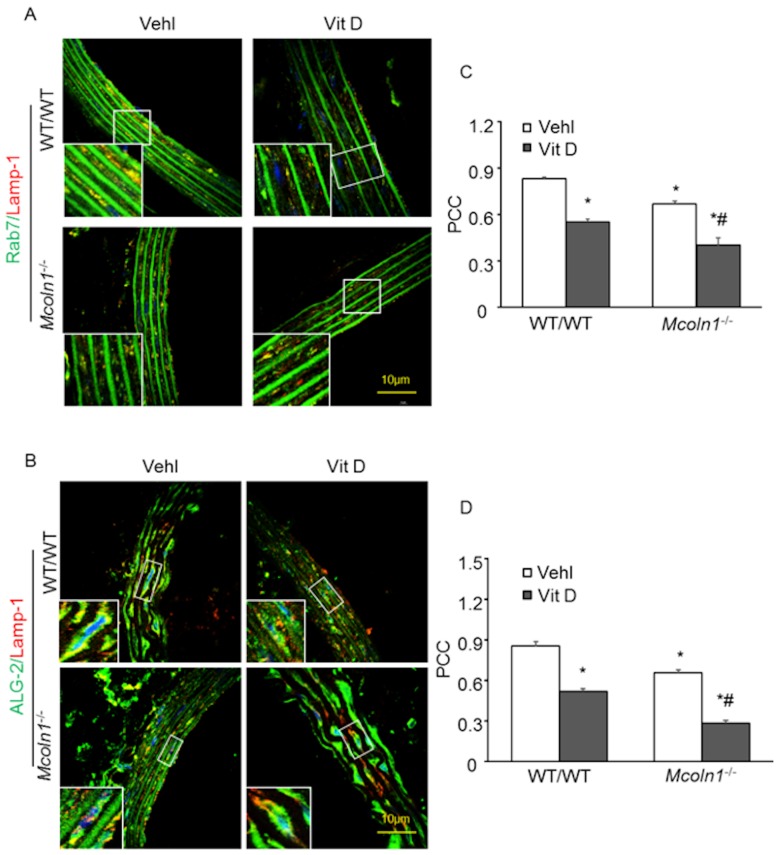
ALG-2 and Rab7 in association with lysosomes promote lysosome coupling in Vit D-treated *Mcoln1*^−/−^ mice. Representative confocal microscopic images depict co-localization of lysosome coupling markers (**A**) Rab7 (green), (**B**) ALG-2 (green), and lysosomes (lysosome marker Lamp-1; red) in aortic medial SMCs was much lower in *Mcoln1*^−/−^ mice than their wild-type littermates receiving Vit D injection (**C**,**D**). The co-localization coefficient (PCC) as shown in the bar graph. Data are shown as mean ± SEM of values. *n* = 5. Vehl: Vehicle; Vit D: vitamin D; PCC: Pearson correlation coefficient. * *p* ˂ 0.05 vs. WT/WT Vehl; # *p* ˂ 0.05 vs. WT/WT Vit D group.

Given the roles of lysosomal TRPML1 and the Ca^2+^ sensor ALG-2 in the process of lysosome coupling to dynein [15], and Rab7-dependent dynein–dynactin recruitment to late endosomes and lysosomes [27], we determined the role of ALG-2 and Rab7 on lysosomal positioning in AMC. We performed confocal microscopy of both ALG-2 and Rab7 and analyzed their co-localization with lysosome marker (Lamp-1). Due to *Mcoln1* gene deletion, decreased co-localization of lysosome marker Lamp-1 (red) with lysosome coupling marker Rab 7 (green) (Figure 6A) and ALG-2 (green) (Figure 6B) was observed in the aortic medial wall of *Mcoln1*^−/−^ mice as compared to wild-type mice, suggesting anterograde movement of lysosomes because of *Mcoln1* gene deficiency. The summarized data are shown as the Pearson correlation coefficient in Figure 6C,D. Hence, these results conclude that ALG-2 and Rab7 in association with TRPML1 promote perinuclear distribution of lysosomes required for the normal degradation process of lysosomes and other cellular processes. 

### 2.5. Mcoln1 Gene Deletion Elevated sEV Secretion by Inhibiting Lysosomes and MVB Interactions in the Arterial Medial Calcification 

**Figure 7:**
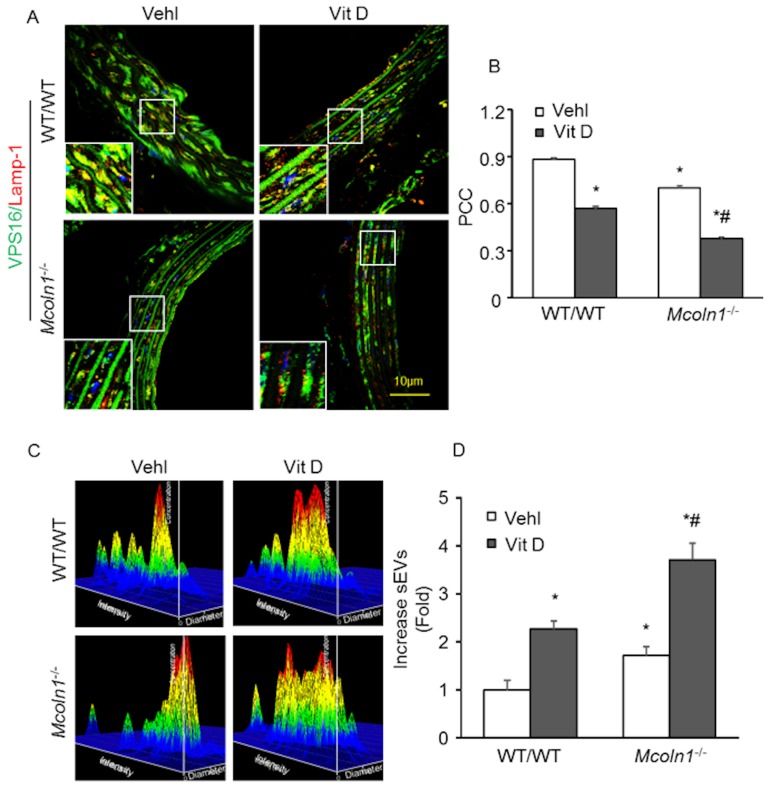
Lysosome–multivesicular body (MVB) interaction and small extracellular vesicle (sEV) release in Vit D-treated *Mcoln1*^−/−^ mice. (**A**) Representative confocal pictures reveal co-localization of MVB (VPS16; green) and lysosome (Lamp-1; red) in aortic medial SMCs (**B**) Bar graph shows significant decrease in co-localization of VPS16/Lamp-1 in Vit D-treated *Mcoln1*^−/−^ mice as compared to wild-type littermates. *n* = 5–6. (**C**) Representative 3D graphs depicting number of sEVs isolated from plasma from wild-type and *Mcoln1*^−/−^ mice with vehicle and Vit D treatment. (**D**) Summarized bar graph shows changes in sEV number (50–150 nm) under vehicle and Vit D treatment. *n* = 5–7. Data are shown as mean ± SEM of values. Vehl: Vehicle; Vit D: vitamin D; PCC: Pearson correlation coefficient. * *p* ˂ 0.05 vs. WT/WT Vehl; # *p* ˂ 0.05 vs. WT/WT Vit D group.

Fusion of late endosomes with lysosomes leads to degradation of the endosomal contents. Moreover, fusion of late endosomes with the plasma membrane releases 40–140 nm vesicles known as exosomes in the outer space of the cell [35]. New molecular mechanisms provided evidence suggesting that SMC-derived extracellular vesicles (EVs), or exosomes, played a crucial role in arterial medial calcification [8]. Under mineral imbalance, VSMCs released calcified EVs that form microcalcification [32]. Therefore, using confocal microscopy we also observed the interaction of lysosomes and MVBs in aortic medial SMCs, which confirms lysosome trafficking and fusion to MVBs. Mice were treated with a high dose of Vit D to cause mineral imbalance. As shown in Figure 7A, co-localization of lysosome marker (Lamp-1; Alex 555, red fluorescence) and MVB marker (VPS16; Alex 488, green fluorescence) produced yellow puncta, indicating the lysosome–MVB interaction. Compared with the control wild-type littermates, Vit D treatment markedly decreased co-localization of Lamp-1 and VPS16 in arterial medial SMCs. *Mcoln1* gene deletion in the mice receiving Vit D injection caused a further decrease in the co-localization of Lamp-1 and VPS16 in aortic medial SMCs, as shown in the bar diagram (Figure 7B). 

**Figure 8:**
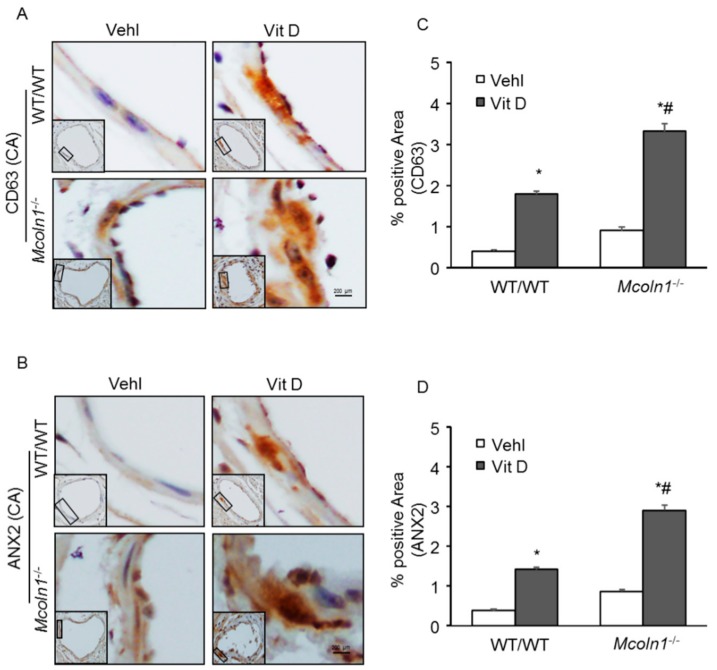
Localization of sEV markers in coronary arterial wall in Vit D-treated *Mcoln1*^−/−^ mice. Representative photomicrographs show immunostaining of sEV makers in coronary arterial wall. (**A**) CD63 and (**B**) AnX2 staining are observed as brown signal in the coronary arterial wall. The summarized bar graph shows % positive area of (**C**) CD63 and (**D**) AnX2 positive staining in coronary arterial wall. Data are shown as mean ± SEM of values. *n* = 5–6. Vehl: Vehicle; Vit D: vitamin D; AnX2: Annexin-II; PCC: Pearson correlation coefficient. * *p* ˂ 0.05 vs. WT/WT Vehl; # *p* ˂ 0.05 vs. WT/WT Vit D group.

Furthermore, by immunohistochemistry, we found significantly increased levels of CD63 (Figure 8A) and annexin-II (AnX2) (Figure 8B) as exosome/sEV markers in the coronary arterial wall of Vit D-treated *Mcoln1*^−/−^ mice compared to the levels of their wild-type littermates. The summarized data are shown as bar diagrams in Figure 8C,D. These results indicate that *Mcoln1* plays an important role in the fusion of lysosomes and MVBs, and *Mcoln1* gene deletion impairs this fusion process and increases sEV release from arterial medial SMCs during AMC.

### 2.6. Role of Mcoln1 in sEV Release in the Arterial Medial Calcification

To test whether *Mcoln1* controls MVB fate and sEV release in the AMC, we performed nanoparticle tracking analysis (NTA) to measure the number of small extracellular vesicles (sEVs) released in the plasma of the mice treated with a high dose of Vit D. Both wild-type and *Mcoln1*^−/−^ mice received Vit D injections to develop AMC. The representative 3-D histograms of vesicles showed the increased release of sEVs (50–150 nm in size) due to *Mcoln1* gene deletion in the plasma of *Mcoln1*^−/−^ mice even without any treatment, as shown in Figure 7C. Additionally, mineral imbalance due to high doses of Vit D-enhanced sEV release in the plasma of *Mcoln1*^−/−^ mice as compared to their wild-type littermates. The bar graph represents the vesicle counts (50–150 nm in size), as shown in Figure 7D. These results suggest that *Mcoln1* is associated with the mechanistic pathway that mediates lysosome positioning and fusion to MVBs and controls MVB fate and sEV release.

### 2.7. Lysosome–MVB Interactions and sEV Release Controlled by TRPML1 Signaling Pathway in Coronary Artery Smooth Muscle Cells (CASMCs)

**Figure 9:**
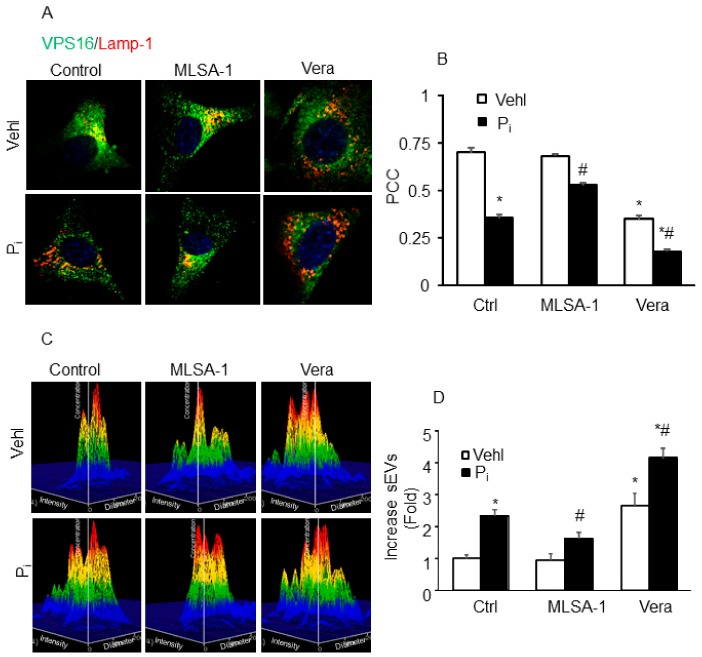
Effects of MLSA-1 and verapamil on lysosome–MVB interactions and sEV release in the wild-type CASMCs in vitro. (**A**) Representative confocal images show co-localization of VPS16 (green) and Lamp-1(red) in CASMCs. (**B**) Bar graph shows ML-SA1 (10 µm) significantly increased and Vera (10µm) caused more decrease in co-localization of VPS16/Lamp-1 in Pi-treated CASMCs. *n* = 3. (**C**) Representative 3-D histograms show sEV release from CASMCs. (**D**) Bar graph shows MLSA-1 decreased sEV release from CASMCs and Vera increased sEV release in these cells. *n* = 3–4. Data are shown as mean ± SEM of values. Phosphate: Pi; Verapamil: Vera. * *p* ˂ 0.05 vs. Ctrl Vehl group; # *p* ˂ 0.05 vs. Ctrl Pi group.

Further using wild-type CASMCs, we tested whether TRPML1 activation or inhibition affects lysosome–MVB interaction and sEV release. Prior treatment with TRPML1 agonist ML-SA1 prominently increased co-localization of VPS16 (MVB marker; green) and Lamp-1 (lysosome marker; red), whereas blockade of TRPML1 by verapamil (Vera) markedly decreased co-localization of VPS16 with Lamp-1 in these cells treated with or without Pi (Figure 9A). Quantitative analysis showed that the PCC of VPS16 with Lamp-1 increased in CASMCs treated with ML-SA1 and decreased by Vera (Figure 9B). Further, we observed that secretion of sEVs (<200 nm) were significantly decreased by ML-SA1 in Pi-treated CASMCs, as shown in Figure 9C,D. However, Vera significantly increased sEV secretion even under vehicle conditions, and caused further increase in Pi-treated CASMCs. These results suggest that *Mcoln1* is important in the regulation of lysosome–MVB interactions and sEV secretion. 

### 2.8. Mcoln1 Gene Deletion Accelerates Arterial Stiffness

**Figure 10:**
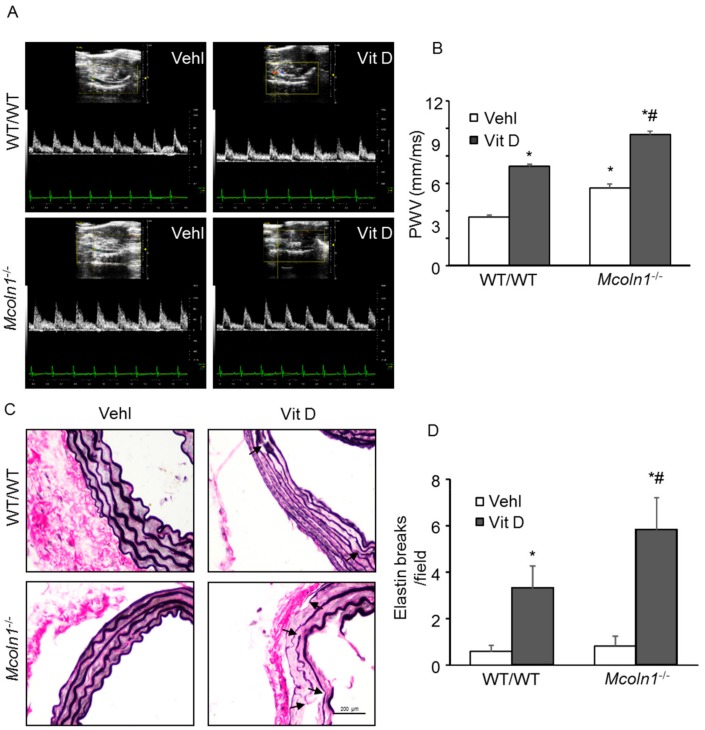
Effect of *Mcoln1* gene deletion on arterial stiffness in Vit D-treated *Mcoln1*^−/−^ mice. (**A**) Representative Doppler images show Doppler velocity signals. (**B**) The summarized bar graph shows significantly increased PWV in *Mcoln1*^−/−^ mice as compared to wild-type littermates under vehicle conditions as well as with Vit D treatment. *n* = 5–6. (**C**) Representative elastin staining images of aortic sections show breaks in elastic fibers (marked by arrows). (**D**) The bar diagram shows number of elastin breaks in wild-type and *Mcoln1*^−/−^ mice receiving Vit D injections. Data are shown as mean ± SEM of values. *n* = 6. Vehl: Vehicle; Vit D: vitamin D. * *p* ˂ 0.05 vs. WT/WT Vehl; # *p* ˂ 0.05 vs. WT/WT Vit D group.

To address whether the *Mcoln1* gene deletion directly contributes to the development of arterial stiffness, we used *Mcoln1*^−/−^ mice. We recently reported that in CASMCs under in vitro conditions, activation of TRPML1 channel enhanced SMC-derived small extracellular vesicles, triggering AMC (unpublished data). PWV is directly correlated with aortic stiffness [36]. Figure 10A shows representative echo images of vehicle and Vit D-treated groups in wild-type and *Mcoln1*^−/−^ mice. As shown in Figure 10B, *Mcoln1* gene deletion increased PWV in *Mcoln1*^−/−^ mice compared with wild-type littermates. To note, *Mcoln1*^−/−^ mice had increased baseline PWV compared to what we usually observe for healthy control mice, suggesting aortic wall stiffening and remodeling in *Mcoln1*^−/−^ even before the development of AMC. Upon receiving high doses of Vit D, we observed a substantial increase in the PWV in the *Mcoln1*^−/−^ mice as compared to their wild-type littermates, further confirming that the *Mcoln1* gene under disturbed blood flow contributes to the arterial stiffness and accelerates the development of AMC.

One of the characteristics of aortic calcification was disorganization of extracellular matrix and elastin degradation. Elastin staining allowed observation of elastin breaks in aortic sections [37]. Vehicle-treated wild-type aortas displayed normal histological architecture associated with lack of calcification and intact elastic lamellae, which were indistinguishable from vehicle-treated *Mcoln1*^−/−^ mice. However, disorganized elastic lamellae with elastin breaks were observed upon Vit D treatment in both wild-type and *Mcoln1*^−/−^ mice, as shown in Figure 10C. Due to *Mcoln1* gene deletion, elastin breaks and disorganization were significantly increased in *Mcoln1*^−/−^ mice as compared to wild-type littermates (Figure 10D), indicating *Mcoln1* gene contributes to arterial stiffness associated with elastin remodeling during the development of AMC.

## 3. Discussion

In the current study, we investigated whether mucolipin-1 (encoded by the *Mcoln1* gene) mediates lysosome positioning leading to sEV release in arterial SMCs, contributing to their phenotype switch and arterial stiffness during the development of AMC. First, we found that *Mcoln1* gene deletion significantly increased arterial medial calcification in mice treated with a high dose of Vit D. Further, we observed markedly increased immunostaining of osteogenic markers such as “bone” transcription factors (e.g., RUNX2) and matrix proteins (e.g., osteopontin) in the arterial medial SMCs of Vit D-treated *Mcoln1*^−/−^ mice as compared to their wild-type littermates. These results showed that osteogenic shifts in arterial medial SMCs with *Mcoln1* gene deletion aid in the development of AMC. We further studied whether *Mcoln1* gene deletion alters lysosome positioning, which may result in the release of sEVs. We observed decreased co-localization of lysosome marker (Lamp-1) with lysosome coupling markers (Rab 7 and ALG-2) in the arterial wall of *Mcoln1*^−/−^ mice as compared to their wild-type littermates, suggesting anterograde movement of lysosomes due to *Mcoln1* gene deletion in *Mcoln1*^−/−^ mice. Also, we observed decreased co-localization of MVBs (VPS16; green) and lysosomes (Lamp-1; red) in the arterial medial SMCs of *Mcoln1*^−/−^ mice as compared to their wild-type littermates associated with increased expression of CD63 and AnX2 (exosome/sEV markers) indicating reduced lysosome–MVB interaction and increased sEV release. In addition, we measured sEVs in the plasma of these mice and found significantly increased plasma sEV in *Mcoln1*^−/−^ mice as compared to their wild-type littermates. Further, we validated lysosome–MVB interaction and sEV release data in vitro in CASMCs using TRPML1 agonist and blocker in wild-type CASMCs to explore the role of TRPML1 pathway. We found that TRPML1 agonist increased co-localization of VPS16-Lamp-1 and decreased sEV release, whereas TRPML1 blocker showed the opposite trend. 

Using a non-invasive ultrasound imaging technique, we observed increased PWV, which is correlated to arterial stiffness due to *Mcoln1* gene deletion in these mice as compared to their littermates. Histopathological architecture showed increased elastin breaks, distorted junctions and formation of solid plates or sheaths in the arterial media of *Mcoln1*^−/−^ mice as compared to their wild- type littermates. Despite our current understanding of the multi-faceted mechanisms regulating AMC, little is known about the lysosomal regulation of sEV release and phenotypic switch of arterial medial SMCs following AMC.

The phenotypic plasticity is an important feature of VSMCs and their contractile phenotype maintains the vascular tone. Upon injury, there is phenotypic transition of these VSMCs from contractile to synthetic. Prolonged exposure of VSMCs to elevated Ca/P_i_ levels induces trans-differentiation of these cells, initiating the formation of osteoblast-like cells, which lead to formation of bone matrix and development of calcification [38,39,40]. These transdifferentiated VSMCs develop features similar to osteoblasts characterized by the increased expression of bone-related genes osteopontin, osteocalcin and RUNX2, and loss of smooth muscle lineage markers such as SM22-*α* [39,41]. Despite improved understanding of VC pathophysiology, the proper mechanistic pathway leading to production of calcifying extracellular vesicles is still poorly understood. In SMCs, imbalanced mineral metabolism resulted in increased secretion of calcifying sEVs, leading to arterial calcification [8]. 

TRPML1, also known as *Mcoln1,* is the primary Ca^2+^ channel in the lysosome [16,42], which is involved in phagosome lysosome fusion [19], membrane fission from endolysosomes, and autophagosome lysosome fusion [43]. Various TRP channels are known to express in ECs and VSMCs [44,45], which play important roles in the regulation of various vascular disorders. Studies have revealed that TRP channels may contribute to vasoconstriction via receptor activation-induced vascular constriction [33,46]. It has been reported that various TRP channels are involved in phenotype switching of SMCs and enhance their proliferation [47,48,49]. Here, we demonstrated that *Mcoln1* gene deletion increased expression of osteogenic markers such as OSP and RUNX2, whereas the expression of contractile marker SM22-*α* was markedly decreased in mice injected with high doses of Vit D, indicating the phenotypic transition of arterial medial SMCs.

Lysosome movement towards the cell periphery is required for exosome secretion, which may contribute to the development of VC [27]. Lysosome distribution throughout the cell is either immobile perinuclear near the microtubule-organizing center (MTOC) or highly dynamic peripheral, reaching as far as the plasma membrane [50]. These spatially distinct pools of lysosomes are involved in different cellular processes to maintain cellular homeostasis through their regular mixing by trafficking and fusion processes [51,52]. Rab 7, a small GTPase, and its downstream effectors are mainly involved in coupling of lysosome to dynein–dynactin. Besides Rab7, the lysosomal Ca^2+^-sensor ALG-2 (also known as PDCD6) regulates coupling of lysosomes to dynein–dynactin. ALG-2 promotes interaction of dynein–dynactin with lysosomal Ca^2+^ channel transient receptor potential mucolipin 1 (*Mcoln1* or TRPML1) to regulate centripetal transport of lysosomes essential for lysosome exocytosis in response to changes in Ca^2+^ levels [15,27]. TRPML1 induces perinuclear redistribution of lysosomes and links with ALG-2, which then causes TRPML1-dependent retrograde transport of lysosome. Earlier, we revealed that activation of TRPML1 channel by sphingolipid sphingosine contributes to lysosome trafficking [31]. In the present study, we found that the *Mcoln1* gene deletion decreased the co-localization lysosome marker (Lamp-1) with lysosome coupling markers (Rab7 and ALG-2) in the aortic medial SMCs during AMC. All these studies support our data, which indicated that *Mcoln1* gene plays a crucial role in the lysosome positioning and its deletion resulted in peripheral distribution of lysosomes, which contributes to the extracellular vesicle release, an important feature in AMC.

In human VSMCs, recent studies revealed that exosomes originated from a subset of late endosomal compartment, MVBs [8]. Lysosomal trafficking and its fusion with late endosomes, MVBs, autophagosomes, and the plasma membrane is controlled by TRPML1, a Ca^2+^-permeable channel [53]. Trafficking of late endosomes, which are generally considered as MVBs, to the plasma membrane results in the release of exosomes [54]. The abovementioned studies provide support to our recent studies and to the present study. Our findings in podocytes demonstrated that inhibition or activation of the TRPML1 channel modulates the interaction of lysosomes and MVBs, thereby regulating the exosome release from these cells [31]. Also, recently, we found that inhibition of TRPML1 channel activation in coronary arterial SMCs due to deficiency of acid ceramidase may lead to the reduction of lysosome–MVB interaction and consequent prolongation of MVB fate, increasing SMC-derived small extracellular vesicle (sEV) release initiating arterial medial calcification (AMC) [55]. Based on these findings, we hypothesized and found that *Mcoln1*^−/−^ mice exhibited abnormal lysosome positioning and increased sEV levels in the arterial medial SMCs, which may contribute to the arterial stiffness during the development of AMC.

However, the study in OP9 cells reported that TRPML1 regulated exosome release by mediating lysosomal exocytosis during adipogenesis. Although the study concluded that depletion of TRPML1 in pre-adipocytes resulted in a marked reduction of surface LAMP1, which indicated that the study focused towards the exosomes present in lysosomes [30]. Also, as mentioned, trafficking of late endosomes to the plasma membrane allows the release of exosomes [54]. However, several studies in drug-resistant cancer cells revealed that TRPMLs regulate release of exosomes protected from degradation within lysosomes through lysosomal exocytosis [56,57]. The reason for these disparities may be the various downstream targets of lysosomal Ca^2+^ such as lysosomal exocytosis by synaptotagmin-VII [58], TFEB activation by calcineurin [59] and ALG-2 required for lysosome motility. Interaction of ALG-2 with the dynactin complex promotes retrograde transport. The particular lysosomal subcellular localization and availability of cofactor or substrate may determine which Ca^2+^ targets are activated for particular Ca^2+^ release events [15]. For instance, lysosomal Ca^2+^ release that is docked to the plasma membrane may activate exocytosis and not the retrograde transport [15]. Therefore, different cells using various mechanistic pathways under diverse pathological conditions may lead to exosome release. Our group revealed that pharmacological inhibition of TRPML1 channel by verapamil significantly increased the exosome release from the podocytes, however its activation in response to ML-SA-1, a potent TRPML channel agonist, markedly decreased exosome release. The study concluded that during podocyte injury, inhibition of TRPML1 channel activity by acid ceramidase deficiency increased podocyte-derived exosome release, which may lead to glomerular damage [31]. Concluding, our data also provide further evidence suggesting that *Mcoln1* gene deletion modulated lysosome–MVB fusion. We observed decreased co-localization of lysosome marker (Lamp-1) with MVB marker (VPS16) due to *Mcoln1* gene deletion in arterial medial SMCs in the development of AMC. 

Next, we investigated whether exosome/sEV secretion triggers VC. Several studies revealed that both intimal and medial VC follow a common mechanistic pathway, namely increased extracellular vesicle secretion, especially exosomes (40–140 nm in size) in interstitial space [11,60]. Using fetuin-A labelling, Kapustin et al. revealed the origin of calcifying exosomes in VSMC [8]. SMC-derived vesicles have increased expression of CD63 and AnX2, identified as exosome markers [10]. However, understanding of the mechanistic pathway mediating exosome biogenesis and secretion in arterial SMCs is still in infancy. In this study, we also observed CD63 and AnX2 (exosome/sEV markers) were significantly increased in the coronary arterial wall of *Mcoln1*^−/−^ mice. Correspondingly, in the plasma of *Mcoln1* gene-deficient mice, we observed increased sEV release, which was markedly enhanced with the Vit D treatment in these mice. These findings suggest that lack of *Mcoln1* gene inhibits perinuclear lysosome trafficking, which is necessary for lysosome-MVB interaction. Impaired lysosome–MVB interaction leads to increased sEV release in arterial medial SMCs. This enhanced sEV release due to *Mcoln1* deficiency may contribute to AMC via cell-to-cell communication. 

Several studies showed that augmented plasma endothelial microparticles (EMPs) are associated with the pathogenesis of cardiovascular diseases [61,62]. Plasma EMPs are identified as independent predictors of long-term cardiovascular events in patients with a high risk for coronary heart disease or diabetes [63,64]. The plasma levels of endothelial microparticles (EMPs) were found to be significantly increased in patients with type 2 diabetes associated with hypertension and arterial stiffness. In addition, they found that various other microparticles (MPs) such as platelet-derived MPs, annexin V + MPs, and leukocyte-derived MPs were significantly higher in patients with diabetes as compared to healthy patients (*p* < 0.05). The study concluded that in diabetics not only increased blood pressure was associated with impaired arterial stiffness but elevated plasma EMPs act as potent contributors in the progression of arterial stiffness in type 2 diabetes [65]. During aging, concentric elastin lamellae develop medial calcification observed as hydroxyapatite deposits, which increase arterial stiffness without the presence of inflammatory cells [66,67]. Elastin degradation is one of the prominent features of aortic calcification and VSMC phenotype is maintained by an intact elastin matrix in vivo [5]. It has been suggested that transient receptor potential (TRP) channels through their Ca^2+^ entry, release, and extrusion mechanisms in membranes regulate VSMC contraction known as vasoconstriction response. Theoretically, Ca^2+^ influx through these channels may directly activate contractile process, but this needs more experimental evidence [68,69]. Moreover, considering the role of transient receptor potential (TRP) channels in VSMC phenotype switching and vasoconstriction, we tried to find out whether *Mcoln1* gene deletion contributes to arterial stiffness in mice [47,48,49]. In this context, we found that *Mcoln1* gene deletion markedly increased Pulse Wave Velocity (PWV) with elastin breaks and disorganization, suggesting that *Mcoln1* deficiency that is associated with vasoconstriction and VSMC phenotypic transition may contribute to the arterial stiffness during the development of AMC.

In summary, the present study revealed that *Mcoln1* gene deletion in mice may play a significant role in both phenotypic maintenance and increased sEV release. *Mcoln1* gene deletion caused abnormal lysosome positioning and increased sEV secretion, which may contribute to the arterial stiffness characteristic of AMC. These findings advance our understanding of the complex mechanistic pathways involved in the development and progression of AMC and can be exploited as a novel therapeutic target for ameliorating this detrimental disease.

## 4. Material and Methods

### 4.1. Reagents and Antibodies 

Cholecalciferol (vitamin D3) (C9756, Sigma Aldrich, St. Louis, MO, USA). Mucolipin1 (SC-26269, Dallas, TX, USA), OSP (ab63856, Abcam, Cambridge, MA, USA), RUNX2 (ab23981, Abcam, Cambridge, MA, USA), SM22-*α* (ab14106, Abcam, Cambridge, MA, USA), VPS16 (Cat. No.17776–1-AP, Protein biotech group, Rosemont, IL, USA), Rab7 (ab137029, Abcam, Cambridge, MA, USA), ALG-2 (PDCD6, A6685, Cummings Park Dr, Woburn, MA, USA.), CD63 (ab216130, Abcam, Cambridge, MA, USA) and annexin-II (AnX2, ab41803, Abcam, Cambridge, MA, USA). Secondary antibodies are Alexa-488 or Alexa-555-labeled (Life technologies, Grand Island, NY, USA). Rat monoclonal anti-mouse Lamp-1 (ab25245, Abcam, Cambridge, MA, USA). Alizarin Red S Solution (TMS-008-C, EMD Millipore. Burlington, MA, USA) was used for detection of arterial medial calcification.

### 4.2. Primary Culture of Mouse CASMCs 

Isolation of mouse CASMCs was described previously [34]. Briefly, CASMCs were cultured in Dulbecco’s modified Eagle’s medium (DMEM, Gibco Gaithersburg, MD, USA), supplemented with 10% fetal bovine serum (Gibco) and 1% penicillin–streptomycin (Gibco Gaithersburg, MD, USA) in humidified 100% air and 5% CO2 mixture at 37 °C. Confluent cells (70%–80%) were treated with or without high phosphate (Pi) (3 mmol/L) [35] and then incubated with TRMPL1 channel agonist, MLSA-1 (10 μM, Sigma-Aldrich Chemicals, St. Louis, MO, USA) and TRMPL1 channel blocker, verapamil (10 μM, Cayman Chemical, Ann Arbor, MI, USA) incubated for 24 h [31,55].

### 4.3. Animal Model 

Male C57 BL/6 J (12–14 weeks old) wild-type and *Mcoln1*^−/−^ mice were employed in the present study. Mice homozygous for the *Mcoln1* gene (*Mcoln1*^−/−^) were generated by crossing mice heterozygous for the *Mcoln1* gene (*Mcoln1*^−/+^) (the heterozygous mice were originally purchased from The Jackson Laboratory, B6.Cg-*Mcoln1* tm1 Sasl/J, Stock No: 027110). The average body weight for the male mice aged 12–14 weeks ranged from ~25–30 g. All mice were grossly normal; the homozygous (*Mcoln1*^−/−^) mice, however, were prone to developing hind-limb paralysis by the age of 28 weeks, and the average life expectancy was 8 months. Heterozygosity and homozygosity within the F1 generation was confirmed using PCR analysis. As seen in Figure 1A, *Mcoln1*^−/−^ mice had one positive PCR product at 750 bp for the mutant *Mcoln1* gene. *Mcoln1*^−/+^ mice had two PCR products: one for the mutant *Mcoln1* gene (750 bp) and one for the WT gene (453 bp). WT/WT mice had only the WT gene (453 bp). All the animal procedures were approved by the Institutional Animal Care and Use Committee of Virginia Commonwealth University (19^th^ Feb 2018) and were registered under protocol number (IACUC, Protocol No. AM10173). Wild-type and *Mcoln1*^−/−^ mice (*n* = 5–6) were further divided into two groups each, and injected with active vitamin D (Vit D) (500,000 IU/Kg/BW/day) or matched vehicle (5% *v*/*v* ethanol) subcutaneously for 3–4 days. Subsequently, animals were sedated with 2% isoflurane through a nose cone after completing a treatment period followed by blood collection and plasma isolation, which was further stored at −80 °C. Animals were sacrificed, heart and aorta were removed, and a part was fixed in 10% buffered formalin for histopathological and immunostaining analysis. Other parts of the heart and aorta were used for frozen tissue slides and stored at −80 °C for dual fluorescence staining and confocal analysis. Vit D-induced mouse model is commonly used for the AMC examination. A high dose of Vit D (500,000 IU/Kg/BW/day) [70] was injected to Normal C57 BL/6 N and *Mcoln1*^−/−^ mice for 3–4 days (*n* = 5–6 per group). Vit D solution was prepared by dissolving vitamin D3 (66 mg) in 200 μL of absolute ethanol and mixing with 1.4 mL of cremophor (Sigma Aldrich, Raleigh, NC, USA), and incubated for 15 min at room temperature, then dextrose solution (750 mg of dextrose dissolved in 18.4 mL of sterilized water) was added and left at room temperature for 15 min. The Vit D solution was stored at 4 °C for further use and made fresh on alternate days. 

### 4.4. Alizarin Red S Staining

Alizarin Red S staining (ARS) has been widely used to detect AMC, as described previously [60]. To process ARS, the artery sections were deparaffinized with alcohol and xylene, followed by three washes of distilled water. Then, the artery sections were stained with 1% Alizarin Red S solution for 5 min and microscopically observed for orange-red color. Further, the sections were dehydrated with acetone for 20 s, followed by acetone–xylene (1:1) solution for 20 s and finally in xylene for 5 min. The sections were then mounted with mounting medium and analyzed under light microscopy. The positive ARS signals showed a reddish color.

### 4.5. Calcium Assay 

Blood concentration of calcium was measured using commercially available kits (ab102505, Abcam, Cambridge, MA, USA) as described by the manufacturer’s protocol. 

### 4.6. Immunohistochemical Analyses

Immunohistochemistry was performed by following the manufacturer’s instructions for vectastain universal kit, peroxidase, RTU (PK-7800, Vector Laboratories, Burlingame, CA, USA). Briefly, paraffin-embedded sections (5 μm) of the aorta were deparaffinized and prepared for antigen retrieval followed by quenching of endogenous peroxidase activity by using 3% H_2_O_2_. For blocking, sections were incubated with 2.5% horse serum for 1 h at room temperature, followed by incubation with primary antibodies overnight at 4 °C against SM22-*α* (1:1500), RUNX2 (1:300), OSP (1:100), CD63 (1:50) and AnX2 (1:50). Subsequently, sections were incubated with biotinylated secondary antibodies and a streptavidin peroxidase complex. Then sections were developed with chromogen 3,3′ Diaminobenzidine (DAB) at RT and rinsed in tap water for 5 min. This rinsing was then followed by counterstaining with hematoxylin for 5 min and dehydration through several washes of alcohol and xylene. Finally, the slides were cleared and mounted with DPX. Image-Pro Plus 6.0 software (Rockvile, MD, USA) was used to calculate the area percentage of the positive staining [71].

### 4.7. Immunofluorescent Staining

To determine the co-localization of various markers in the arterial medial wall, indirect immunofluorescence staining was performed. In this double-layer method, the unconjugated primary antibody binds to the target and fluorochrome-conjugated secondary antibody directed against the primary antibody. Briefly, frozen aortic sections were fixed in acetone, and then sections were incubated in a blocking buffer for 1 h. Subsequently, aortic sections were incubated with diluted primary antibodies of VPS16 (1:300), Rab7 (1:50), ALG-2 (1:100), and (Lamp-1) (1:200) overnight at 4 °C, followed by fluorochrome-conjugated (either Alexa-488 or Alexa-555-labeled) secondary antibody incubation for 1 h at room temperature. Sections were then rinsed in washing buffer. Finally, sections were mounted with DAPI mounting medium, and staining was examined under a confocal laser scanning microscope (Fluoview FV1000, Olympus, Tokyo, Japan). The rate of co-localization of Lamp-1 with RAB7, ALG-2, VPS16 in the aortic medial SMCs was expressed by Pearson’s Correlation Coefficient (PCC) using Image-Pro Plus 6.0 software (Media Cybernetics, Bethesda, MD, USA). PCC is determined by correlation coefficient, *r* (*r* = −1 to 0), as mentioned previously [71,72]. 

### 4.8. Nanoparticle Tracking Analysis (NTA) 

Nanoparticle Tracking Analysis (NTA) was used to analyze the sEV size and concentration in the plasma of mice using the light scattering mode of the NanoSight LM10 (Nano Sight Ltd., Enigma Business Park, Amesbury, United Kingdom). Plasma was prepared by high-speed centrifugation from anticoagulated blood of mice followed by isolation of sEVs from plasma. Briefly, plasma/cell culture supernatant was centrifuged at 300 g at 4 °C for 10 min to remove detached cells or debris and then filtered through 0.22 μm filters to remove apoptotic bodies and microvesicles. Then subjected to ultracentrifugation at 100,000× *g* for 90 min at 4 °C (Beckman 70.1 T1 ultracentrifuge rotator) [73]. To perform NTA, the sEV pellet was diluted in PBS. Each diluted sample was captured in 5 frames (30 s each) with background level 10, camera level 12, and shutter speed 30. The captured video was analyzed with NTA software (Version 3.2 Build 16), and an average particle size distribution graph was plotted with PRISM software (GraphPad, San Diego, CA, USA) [8]. 

### 4.9. Real Time-PCR Studies 

The mRNA levels of *TRPML1* (also known as mucolipin 1 gene), OSP and RUNX2 were determined from aorta in the wild-type and *Mcoln1*^−/−^ mice by RT-PCR. The following mouse-specific primers were used: *β*-actin, sense 5′-TCGCTGCGCTGGTCGTC-3′ and antisense 5′-GGCCTCGTCACCCACATAGGA-3′; mucolipin-1, sense 5′-ATTTGCTTTTGCCTGTTTGG-3′ and antisense 5′-CTCCATCGTCATCATCATCG--3′; OSP, sense 5′-ATTTGCTTTTGCCTGTTTGG-3′ and antisense 5′-CTCCATCGTCATCATCATCG--3′; RUNX2 sense 5′-CCAGATGGGACTGTGGTTACC-3′ and antisense 5′-ACTTGGTGCAGAGTTCAGGG-3′. *β*-Actin was used as an internal control to normalize the expression of mRNA. Fold changes were calculated as follows: 2^−ΔΔ threshold cycle^ [74]. 

### 4.10. Western Blot Analysis

Briefly, SDS-PAGE was used to resolve equal amounts of protein isolated from the aorta along with the molecular weight marker, transferred to PVDF membrane. Milk (5%) was used for blocking the membrane, followed by incubation with respective primary antibodies rabbit polyclonal to SM22-*α* (1:10,000, abcam, Cambridge, MA, USA), rabbit polyclonal to RUNX2 (1:1000, abcam, USA) and rabbit anti-*β*-actin (1:10,000, Santa Cruz Biotechnology, Dallas, TX, USA) overnight at 4 °C. Next day incubated with donkey anti-rabbit-HRP IgG (1:5000, Santa Cruz Biotechnology, Dallas, TX, USA) for 1 h at room temperature. Finally, bands were detected by chemiluminescence technique using LI-COR Odyssey Fc and the band intensity of target proteins was normalized to *β*-actin and calculated with Image J software version 1.44 p (NIH, Bethesda, MD, USA) [71].

### 4.11. Non-Invasive, in Vivo Measurement of Aortic Pulse Wave Velocity (PWV) 

Pulse Wave Velocity (PWV) was used to measure arterial stiffness post-Vit D treatment by using a high-resolution Doppler ultrasound instrument (Vevo2100, Visual-Sonics, Toronto, ON, Canada) [75]. Briefly, animals were anesthetized with 2% isoflurane and then mounted on a temperature-controlled platform where the body temperature of mice was maintained at 37 °C to remove the movement artifacts. Different parameters, such as an electrocardiogram (ECG), heart rate (HR), and respiratory rate were then observed. In addition, heart rate (HR) was maintained around 450 bpm by adjusting isoflurane concentration to 1.5%. Flow wave Doppler measurement of aortic outflow was captured with a 6 mm, 30 MHz pulsed Doppler probe with a pulse repetition frequency of 40 kHz. Aortic outflow profiles were captured longitudinally at two locations, one proximal and the other distal to the heart. Heart rate was similar in both proximal and distal point measurements. PWV was estimated at both the proximal and distal locations from the peak of the ECG R wave to the foot of the flow waves for 5 replicates in each mouse. PWV (mm/ms) was calculated using the TT(Δt) and the distance between the measurement sites (Δd), where TT (Transit time) is the difference between the time of arrival at proximal and distal points [75].

### 4.12. Elastin Staining

Elastin staining or Verhoeff’s Van Gieson stain was used to stain elastic fibers in aortic tissue sections. Staining was performed by following manufacturer’s instructions (ab150667, Abcam, Cambridge, MA, USA). Briefly, sections were deparaffinized, then hydrated in distilled water. Sections were then incubated in elastin stain solution for 15 min, rinsed in running tap water to remove excess stain. Further, sections were dipped in differentiating solution 15–20 times, rinsed in tap water, and checked under the microscope for proper differentiation. Then, sections were incubated in sodium thiosulfate solution for 1 min, rinsed in running water, followed by staining of sections using Van Gieson solution for 2–5 min. Lastly, sections were washed in 95% alcohol 2 times, dehydrated in absolute alcohol, and mounted in synthetic resin. 

### 4.13. Statistical Analysis

All of the values are expressed as mean ± SEM. One-way and two-way ANOVA was used to examine the significant differences among multiple groups, followed by a Tukey’s or Duncan’s test. *p* < 0.05 was considered statistically significant.
